# Graduate Study on the Continent

**Published:** 1908-10-10

**Authors:** 


### GRADUATE STUDY ON THE CONTINENT.

### GRADUATE STUDY IN AUSTRIA.—I.

There are two aspects of graduate study, namely, the local and the general. The first may be defined as the realisation by the profession locally that graduate study is a necessity for development and progress. It is seen in the recent graduate study movement in Germany, that has had such brilliant success, and that has placed the German universities easily at the head of graduate centres on the Continent. But this aspect is, after all, a purely local one. The development is the outcome of local effort and local progressiveness. The aim is, first and foremost, to benefit the local practitioner. This is only as it should be. It is the duty of a country to care, first of all, for its own doctors, and only secondly to put the best of its clinical material at the disposal of their foreign colleagues. In all our articles on post-graduate study on the Continent we have fully admitted this fact, and have repeatedly laid stress on the fact that when attending post-graduate courses at a Continental centre the student should regard himself as a privileged guest, not as a customer who has entered into a commercial transaction to obtain the best value for the least expenditure of money. We have thought it advisable to give particulars, briefly and clearly, of the development of local post-graduate institutions; in the first place to show how far ahead of us Germany is in the matter of facilitating graduate study for her own practitioners, and secondly in order that these particulars may be of help and use to such English students as may desire to join German post-graduate courses. In dealing with Austria we shall follow on the same line, but the summary will be a much shorter one. For essentially Austria differs from Germany in this respect, that it offers practically no more facilities to its own students than it does to the foreign graduate. It is true that for many years regular practitioners' courses have been held during the vacations at Budapest, and lately, following the German example, local energies have crystallised into action at such centres as Vienna and Prague, but the time has not yet arrived when one can honestly write about the advance of graduate study in Austria for Austrian practitioners.

Far otherwise, however, is what may be called the general aspect of the matter—the aspect that chiefly interests the foreign graduate. The man who comes to the Continent does not ask himself "What facilities exist for the local practitioner in the country whither I am going?" His interest is a more selfish one, and this is only natural.

We may preface all our chapters by stating that for the foreign graduate who is willing to spend some time and to devote himself rather more than cursorily to medical or surgical study, Austria offers more opportunities than does Germany. This is especially the case so far as practical work is concerned. At present, for instance, it is impossible for an English or American graduate, who has not passed the German State examination, allowing him to practise as a physician and surgeon in Germany, to hold a post, even as a volunteer assistant, at any German clinic. This new rule came into force last May, and deprives graduate students of the benefits which such an assistantship undoubtedly conferred. Before it was enforced it was not unusual for English practitioners, known to the chiefs of a clinic, to be appointed as assistants, and we know of many instances where such men have done good work and used to the full the opportunities that came to them in that capacity. In Austria there is as yet no such rule. The foreign student, provided he is qualified and in a position to practise in his native country, may hold a responsible position at a clinic just as formerly he was able to act as volunteer assistant at a German clinic. This is possible because legally the responsible chief of a clinic is the professor in charge of it, who must, of course, be a State-licensed practitioner. With us, where the house surgeon or house physician must be a licensed practitioner, the case is somewhat different, for the house surgeon or house physician is directly responsible for the cases under his charge. In Austria the distinction between hospital and private practice is, perhaps rightly, not made to rest on the same footing, and up to a certain extent the assistants are purely assistants whose certificates and doings are tacitly supposed to have been verified and ratified by their chief. Not that the Austrian assistant is a person of less importance or with less responsibility than a house surgeon or house physician with us. On the contrary, he is much more responsible, much more important, and has much more chance for practical work. Usually the number of beds under his charge is large—rarely less than 30—and the opportunities for operative work, in the case of the surgical assistants, are exceptionally good. For this reason, then, the foreign graduate will find Austria attractive, provided he has the time and the knowledge of the language necessary to justify him in applying for an assistantship.

Again, the amount of clinical material available for study at Austrian hospitals is unusually rich and varied. There are far too few hospitals for the large population of the chief towns. Thus Vienna suffers from a plethora of material, although its hospital accommodation per head of population is far beyond that of any other Austrian city. Concentration and centralisation are more apparent in Austria than in Germany so far as hospitals are concerned. The clinics are all large; some of them, as the medical clinic at Vienna, the surgical clinic at Graz, and the gynæcological at Prague, exceptionally so. There is, generally speaking, more scope for individual work, because the number of assistants (with the exception of Vienna) is unusually limited; there is more work than the staff can calmly deal with. This is especially the case at the less well-known centres such as Innsbruck, Graz, and Budapest. The material is drawn from a very wide field, and the larger clinics serve not only their own local centre but outlying districts, and attract even patients from the border provinces of Russia, Roumania, and Servia.

Another point that adds to the attractions of study at an Austrian centre is that the teaching is everywhere good. In Germany it is subject to fluctuations, and at one centre the teaching may be exceptionally fine, while again at another it is open to serious criticism. The teachers at the clinics are for the most part men who have a recognised reputation as original workers in their special branch. At every centre there are men who have a more than local reputation and who are, if one may say so, of international celebrity. This is especially true in surgery. In pure medicine, in which Austria at one time took the lead, she is at present surpassed by Germany, and in gynæcology she can no longer hold her own, but in surgery she still deserves attention as being in the front rank. One has only to look at the lists of names in the various calendars to acknowledge this. The student will find it a great advantage, in one respect, to have a limited choice of centres. In Germany there are too many attractions; with forty centres to choose from the student may well hesitate before he attaches himself to one. In Austria, however, there is a concentration which, from the foreign graduate's point of view, is eminently satisfactory. The choice lies between Vienna, Innsbruck, Graz, Prague, and Budapest, and at a pinch the student may spend part of his time at each of these. For the man who is chiefly interested in only one branch of medicine that is a course we should heartily recommend.

Except at Vienna, the English graduate student is not very well known in Austria. Few men go farther afield. The majority remain in the capital, and Austrian medicine and Austrian methods, to them, are purely Vienna medicine and Vienna surgery. When the student, therefore, goes to another centre he will not find any "courses" in the Vienna sense of the word. That is to say, there will not be a group of English-speaking students who regularly attend so many times a week to get a "lesson" from one or other of the recognised graduate study professors, and pay for this privilege a sum varying from 20 to 100 kronen. He will find that he can attend excellent lectures, fully equal to, and in many respects better than, those which he can obtain at the capital, on payment of the ordinary students' fees. If he wishes to take a course, he can either arrange privately with the professor or assistants, or he can take a vacation course such as is given for the benefit of the junior qualified assistants. These courses, especially those in operative surgery, we can thoroughly recommend. Usually, also, one or other of the docenten is willing to "read" on some special subject if he can obtain sufficient members to form a class. In the smaller centres this is not always possible, and if the student is bent on having a special course we should strongly advise him to beat up a party from the large number of men at Vienna who are always keen on courses, and who do not know the attractions of such places as Graz and Budapest, and then to fix on some centre and arrange for a course with the professor or docent. There will not be much difficulty in such an arrangement. All the professors express surprise that foreign students flock almost exclusively to Vienna, and declare themselves perfectly willing to give similar courses to those which can be obtained in the better-known centre if they can get a class together. Our experience at Prague, Innsbruck, and Graz has convinced us that these centres only need to be better known to English-speaking students to rival Vienna in popularity as graduate centres.

We may add to the list of attractions by pointing out that life at an Austrian centre has many advantages from a purely social point of view. All the chief centres are situate in districts which rank among the most picturesque and beautiful in Europe. For the sportsman, the mountaineer, and for the man who wants to combine business with pleasure they offer more scope than any German centre does. Living is, with the exception of Vienna, generally cheap, although in this respect the comparison will not be absolutely against the German centre. Courses, where given, are relatively cheaper than in Germany, and additional expenses (again excepting Vienna and possibly also Budapest) also compare favourably with those at a German centre. Having put the advantages of study in Austria, we may as briefly deal with its drawbacks. One of these, and perhaps the greatest, is the want of good medical libraries and reading-rooms. Even at Vienna, which is by far the best provided in this respect, the facilities for reference to literature leave much to be desired, while at the smaller centres they are not to be compared with those of a large German centre. There is, of course, always the possibility of sending to the larger centres for the wished-for literature, but that is a method which has its obvious drawbacks. Especially will the student feel the want of such facilities when he wishes to refer to English or American literature. Another drawback is that one centre, Vienna, is overcrowded with English-speaking students. This does not, of course, apply to other centres, as we shall show in succeeding articles. Then, again, the pathological and clinical collections available for study are usually badly arranged and by no means so convenient as in Germany. But all these disadvantages can scarcely outweigh the undoubted attractions. Austria is a country that should appeal to the graduate student. It is a country where he can work more quietly than he can anywhere else, where he can command almost unlimited material, and where he can obtain as good clinical instruction as in any country in the world.

